# Novel Mutations in X-Linked, *USP26*-Induced Asthenoteratozoospermia and Male Infertility

**DOI:** 10.3390/cells10071594

**Published:** 2021-06-25

**Authors:** Chunyu Liu, Ying Shen, Qunshan Shen, Wen Zhang, Jiaxiong Wang, Shuyan Tang, Huan Wu, Shixiong Tian, Jiangshan Cong, Xiaojin He, Li Jin, Feng Zhang, Xiaohui Jiang, Yunxia Cao

**Affiliations:** 1 Obstetrics and Gynecology Hospital, NHC Key Laboratory of Reproduction Regulation (Shanghai Institute for Biomedical and Pharmaceutical Technologies), Fudan University, Shanghai 200011, China; 17111250002@fudan.edu.cn (C.L.); sy_tang05@outlook.com (S.T.); 20110880002@fudan.edu.cn (S.T.); 19111250001@fudan.edu.cn (J.C.); lijin@fudan.edu.cn (L.J.); zhangfeng@fudan.edu.cn (F.Z.); 2 Shanghai Key Laboratory of Female Reproductive Endocrine Related Diseases, Shanghai 200011, China; 3 Department of Obstetrics/Gynecology, Key Laboratory of Obstetric, Gynecologic and Pediatric Diseases and Birth Defects of Ministry of Education, West China Second University Hospital, Sichuan University, Chengdu 610041, China; yingcaishen01@163.com; 4 Reproductive Medicine Center, Department of Obstetrics and Gynecology, The First Affiliated Hospital of Anhui Medical University, Hefei 230022, China; shenqunshan520@163.com (Q.S.); wu_huan5@163.com (H.W.); hxj0117@126.com (X.H.); 5 NHC Key Laboratory of Study on Abnormal Gametes and Reproductive Tract, Anhui Medical University, Hefei 230032, China; 6 Key Laboratory of Population Health Across Life Cycle, Anhui Medical University, Ministry of Education of the People’s Republic of China, Hefei 230032, China; 7 Fudan University Pudong Medical Center, Shanghai Key Laboratory of Medical Epigenetics, Institutes of Biomedical Sciences, Department of Systems Biology for Medicine, School of Basic Medical Sciences, Fudan University, Shanghai 200032, China; wenz@fudan.edu.cn; 8 Center for Reproduction and Genetics, The Affiliated Suzhou Hospital of Nanjing Medical University, Suzhou Municipal Hospital, Suzhou 215002, China; wjx8705@163.com; 9 Reproductive Andrology/Human Sperm Bank, West China Second University Hospital, Sichuan University, Chengdu 610041, China

**Keywords:** male infertility, asthenoteratozoospermia, *USP26*, intracytoplasmic sperm injections

## Abstract

Male infertility is a multifactorial disease with a strong genetic background. Abnormal sperm morphologies have been found to be closely related to male infertility. Here, we conducted whole-exome sequencing in a cohort of 150 Han Chinese men with asthenoteratozoospermia. Two novel hemizygous mutations were identified in *USP26*, an X-linked gene preferentially expressed in the testis and encoding a deubiquitinating enzyme. These *USP26* variants are extremely rare in human population genome databases and have been predicted to be deleterious by multiple bioinformatics tools. Hematoxylin-eosin staining and electron microscopy analyses of the spermatozoa from men harboring hemizygous *USP26* variants showed a highly aberrant morphology and ultrastructure of the sperm heads and flagella. Real-time quantitative PCR and immunoblotting assays revealed obviously reduced levels of *USP26* mRNA and protein in the spermatozoa from men harboring hemizygous deleterious variants of *USP26*. Furthermore, intracytoplasmic sperm injections performed on infertile men harboring hemizygous *USP26* variants achieved satisfactory outcomes. Overall, our study demonstrates that *USP26* is essential for normal sperm morphogenesis, and hemizygous *USP26* mutations can induce X-linked asthenoteratozoospermia. These findings will provide effective guidance for the genetic and reproductive counseling of infertile men with asthenoteratozoospermia.

## 1. Introduction

Sperm biogenesis is a complex biological process that requires accurate spatio-temporal regulation of a list of specific genes; the absence of any important gene will lead to abnormal spermatogenesis [1]. Asthenoteratozoospermia, an important factor leading to male infertility, has been defined as a disorder of genetic origin, and recurrent mutations have been identified in several specific phenotypes, including macrozoospermia, globozoospermia, and multiple morphological abnormalities of the flagella (MMAF). For example, homozygous mutations of *AURKC* were identified to be responsible for most cases of macrozoospermia [2]. Gene defects in *SPATA16* and *DPY19L2* were found to be associated with typical globozoospermia [3,4]. Furthermore, 22 MMAF-associated genes have been reported since the initial identification of *DNAH1* [5,6,7,8,9,10]. All of these findings indicated the genetic heterogeneity of asthenoteratozoospermia and the potential involvement of other pathogenic factors.

Ubiquitination is an important biological process that controls the stability and degradation of cellular proteins [11]. The addition of ubiquitin to substrate proteins, mediated by the relayed reactions of ubiquitin-associated enzymes, can promote the degradation of target proteins in proteasomes [12]. In contrast, the removal of ubiquitin from substrate proteins (also called deubiquitination), catalyzed by ubiquitin-specific peptidases (USPs; also known as deubiquitinases (DUBs)), can prevent target proteins from being degraded [12]. The balance between ubiquitination and deubiquitination is essential for the correct completion of spermatogenesis, as it regulates the biological activity, stability, or subcellular localization of related proteins [13,14,15]. As a member of the USP family, numerous nucleotide variations in *USP26* have been identified in infertile men [16,17,18]. However, conflicting results obtained by Luddi et al. also revealed a nonsense mutation in *USP26* in a normospermic man, making the association of variations in *USP26* with male infertility unclear [19].

In this work, we analyzed genetic data obtained by whole-exome sequencing (WES) from a large cohort of 150 asthenoteratozoospermia-affected Chinese men, and identified two patients harboring hemizygous variants in *USP26*, a gene specifically expressed in the testis and encoding an important deubiquitinating enzyme. The subjects harboring hemizygous variants in *USP26* displayed reduced progressive sperm motility and multiple malformations in sperm morphology. Notably, a good pregnancy outcome was acquired by intra-cytoplasmic sperm injection (ICSI) treatment, using the spermatozoa from the two men harboring hemizygous *USP26* variants. These findings suggest that a deficiency in USP26 can cause severe asthenoteratozoospermia, mainly manifesting as multiple malformations in both the sperm heads and flagella.

## 2. Materials and Methods

### 2.1. Subjects and Clinical Investigation

A cohort of 150 subjects affected by asthenoteratozoospermia was enrolled from the First Affiliated Hospital of Anhui Medical University, the Human Sperm Bank of West China Second University Hospital of Sichuan University, and the Affiliated Suzhou Hospital of Nanjing Medical University. All the recruited individuals displayed isolated infertility without obvious primary ciliary dyskinesia-related symptoms, such as bronchitis, sinusitis, otitis media, or pneumonia [20]. A clinical investigation suggested that all the probands in this study displayed normal male external genitalia, bilateral testicular sizes, hormone levels, and secondary sexual characteristics. All individuals had normal somatic karyotypes (46, XY), with no large-scale deletions in the Y chromosome. Informed consent was obtained from all of the subjects and their family members participating in the study. This study was approved by the institutional review boards at Fudan University, the First Affiliated Hospital of Anhui Medical University, West China Second University Hospital of Sichuan University, and the Affiliated Suzhou Hospital of Nanjing Medical University.

### 2.2. Whole-Exome Sequencing and Bioinformatic Analysis

Whole-exome sequencing (WES) of the 150 subjects was performed according to a previously described protocol [21]. In brief, genomic DNA was isolated from peripheral blood samples of human subjects by the DNeasy Blood and Tissue Kit (QIAGEN, Germany, 51106). Then, 1 µg of genomic DNA was used to enrich the human exome by using the AIExome Enrichment Kit V2 (iGeneTech, Beijing, China), and was sequenced on the Novaseq 6000 platform (Illumina, San Diego, CA, USA). The obtained original data were mapped to the human genome assembly (GRCh37/hg19) by Burrows-Wheeler Aligner (BWA) software, and Picard software was used to evaluate the quality of variants and remove PCR duplicates [22]. ANNOVAR software was further used for functional annotation with information from OMIM, Gene Ontology, KEGG Pathway, SIFT, PolyPhen-2, MutationTaster, 1000 Genomes Project, and gnomAD [23]. Nonsense, frameshift, and essential splice-site variants were preferred. Missense variants predicted to be deleterious simultaneously by the bioinformatic tools of SIFT, PolyPhen-2, and/or MutationTaster were also included for further evaluation. Sanger sequencing was conducted for variant verification, and the primers are listed in Table S1.

### 2.3. Structural Modeling for USP26 and Its Mutants

The sequences of USP26 and its mutant Arg825Gly were submitted to the Swiss Model web server (https://swissmodel.expasy.org/ (accessed on 17 March 2021)) to perform homology modeling. The fragments around Arg825 and Arg825Gly were selected for the search model, and the final model was built using default settings (the template identity was about 31%). The full-length sequences of USP26 and its mutants Arg825Gly and Asn799Ser were submitted to the I-TASSER server (http://zhanglab.ccmb.med.umich.edu/I-TASSER (accessed on 25 January 2021)) to carry out structure prediction. Five models were compared, and the best model with common good sharp and a regular local structure was selected for further analysis. The local structure around Arg825, modeled by the Swiss Model server, was similar to the structure model predicted by the I-TASSER server (superposition RMSD~2.3 Å).

### 2.4. Semen Characteristics Analysis

Semen samples were obtained by masturbation after a period of 2 to 7 days of sexual abstinence, and analyzed in the source laboratories during a routine biological examination according to the Fifth World Health Organization (WHO) guidelines. Analyses of semen volume, sperm concentration, and motility were replicated three times. Sperm morphology was assessed with hematoxylin and eosin (H&E) staining and scanning electron microscopy (SEM), including six categories: thin heads, absent, short, bent, coiled flagella, and flagella of irregular caliber. For each subject, at least 200 spermatozoa were examined to evaluate the rates of morphologically abnormal spermatozoa.

### 2.5. Electron Microscopy Evaluation

For electron microscopy evaluation, spermatozoa were prepared according to a previously described protocol [10]. For the SEM assay, sperm specimens were deposited on poly-L-lysine-coated coverslips, immersed in 2.5% glutaraldehyde, rinsed in 0.1 mol/L phosphate buffer, and post-fixed in osmic acid. Then, the sperm specimens were progressively dehydrated with an ethanol and isoamyl acetate gradient and dried by a CO_2_ critical-point dryer (Eiko HCP-2, Hitachi). Next, the specimens were mounted on aluminum stubs, sputter-coated using an ionic sprayer meter (Eiko E-1020, Hitachi), and analyzed via SEM (Stereoscan 260) under an accelerating voltage of 20 kV. For transmission electron microscopy (TEM) assay, semen samples were washed and immersed routinely. Then, dehydration was conducted using graded ethanol (50%, 70%, 90%, and 100%) and 100% acetone, followed by infiltration with 1:1 acetone and SPI-Chem resin overnight at 37 °C. After infiltration and embedding in Epon 812, the specimens were sliced with ultra-microtome and stained with uranyl acetate and lead citrate. Then, the slices were observed and photographed via TEM (TECNAI-10, Philips) with an accelerating voltage of 80 kV.

### 2.6. Real-Time Quantitative PCR(RT-qPCR)

The total RNA of human spermatozoa was extracted using the Allprep DNA/RNA/Protein Mini Kit (QIAGEN), and approximately 1 µg of obtained RNA was converted into cDNAs using HiScript II Q RT SuperMix for quantitative PCR (Vazyme). The obtained cDNAs were individually diluted 5-fold to be used as templates for the subsequent real-time fluorescence quantitative PCR, with AceQ qPCR SYBR Green Master Mix (Vazyme) on a CFX Connect Real-Time PCR Detection System. *GAPDH* was used as an internal control, and the primers for real-time quantitative PCR are listed in Table S2. The expression of mRNA was quantified according to the 2^−^^△△Ct^ method.

### 2.7. Immunoblot Analysis

The proteins of human spermatozoa were extracted using Minute^TM^ Total Protein Extraction Kit for Animal Cultured Cells and Tissues (Invent), and were separated by 10% sodium dodecyl sulfate polyacrylamide gel electrophoresis (SDS-PAGE), followed by transferal to a polyvinylidene difluoride (PVDF) membrane (Millipore) for immunoblot analysis. Membranes were blocked in 5% non-fat milk for 1 h at room temperature before incubation overnight at 4 °C with the following primary antibodies: rabbit polyclonal anti-USP26 (NBP2-93692, Novus, 1:1000) and HRP-conjugated beta actin (HRP-60008, Proteintech, 1:2000). After being washed in TBST (Tris-buffered saline with Tween-20) three times, the membranes were further incubated with HRP-conjugated anti-Rabbit IgG antibody (M21002, Abmart, 1:2500) for 1 h at room temperature. After washing three times in TBST, ChemistarTM High-sig ECL Western Blotting Substrate (Tanon) was used to detect the immunoreactive protein bands by Tanon 5200.

### 2.8. Immunofluorescence Analysis

For immunofluorescence staining, the sperm cells were washed and fixed in 4% paraformaldehyde for 30 min at room temperature, followed by two washes with PBS, and smeared onto slides pre-coated with 0.1% poly L-lysine (Thermo Scientific). Then, the slides of sperm cells were blocked in 10% donkey serum for 1 h at room temperature, before being incubated overnight at 4 °C with the following primary antibodies: rabbit polyclonal anti-USP26 (NBP2-93692, Novus, 1:100), and monoclonal mouse anti-a-tubulin (T9026, Sigma, 1:500). Next, washes were performed using PBS with 0.1% (*v*/*v*) Tween20, followed by 1 h incubation at room temperature with highly cross-adsorbed secondary antibodies Alexa Fluor 488 AffiniPure Donkey anti-Mouse IgG (34106ES60, Yeasen, 1:1000) and Cy3-conjugated AffiniPure Goat Anti-rabbit IgG (111-165-003, Jackson, 1:4000). Images were captured with a confocal microscope (Zeiss LSM 880).

## 3. Results

### 3.1. Identification of Hemizygous USP26 Variants in Men with Asthenoteratozoospermia

**Figure 1:**
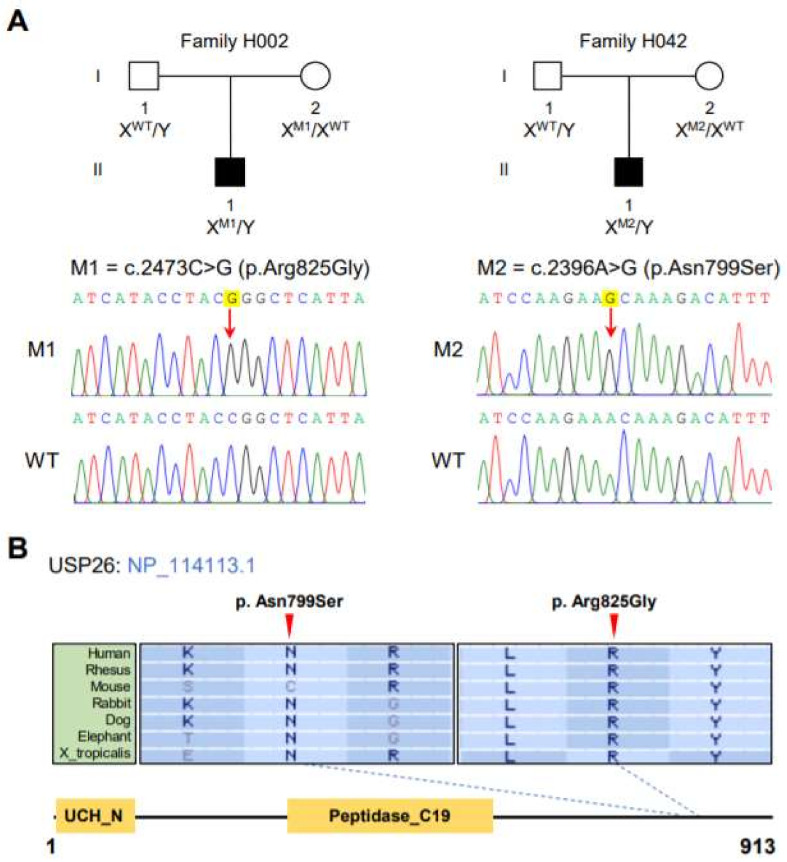
Identification of hemizygous variants of X-linked *USP26* in men with asthenoteratozoospermia. (**A**) Pedigrees of two families affected by hemizygous *USP26* variants. Black-filled squares indicate the male individuals with asthenoteratozoospermia. Sanger sequencing results are shown below the pedigrees. The variant positions are indicated by red arrows. WT, wild type. (**B**) Variant locations and phylogenetic conservation of the mutated residues in the USP26 protein. The NCBI reference sequence number for the USP26 protein is NP_114113.1. The orange boxes indicate different domains, as described by the NCBI browser. The UCH_N, N-terminal of ubiquitin carboxyl-terminal hydrolase 37; Peptidase_C19, Peptidase C19 contains ubiquitinyl hydrolases.

In this study, whole-exome sequencing analyses were performed on the cohort of 150 subjects affected by asthenoteratozoospermia, according to a previously described protocol [21]. After applying stringent bioinformatic analyses, we identified two men harboring hemizygous missense variants in *USP26* (MIM: 300309): c.2473C>G (p. Arg825Gly) in subject H002 II-1 and c.2396A>G (p. Asn799Ser) in subject H042 II-1 (Figure 1A). Subsequent Sanger sequencing revealed that these hemizygous *USP26* variants were inherited from their heterozygous maternal carriers (Figure 1A). Both of the hemizygous variants in *USP26* are absent or extremely rare in human genome datasets, and have been predicted to be damaging through the use of the PolyPhen-2, SIFT, M-CAP, and CADD tools (Table 1).

**Figure 2:**
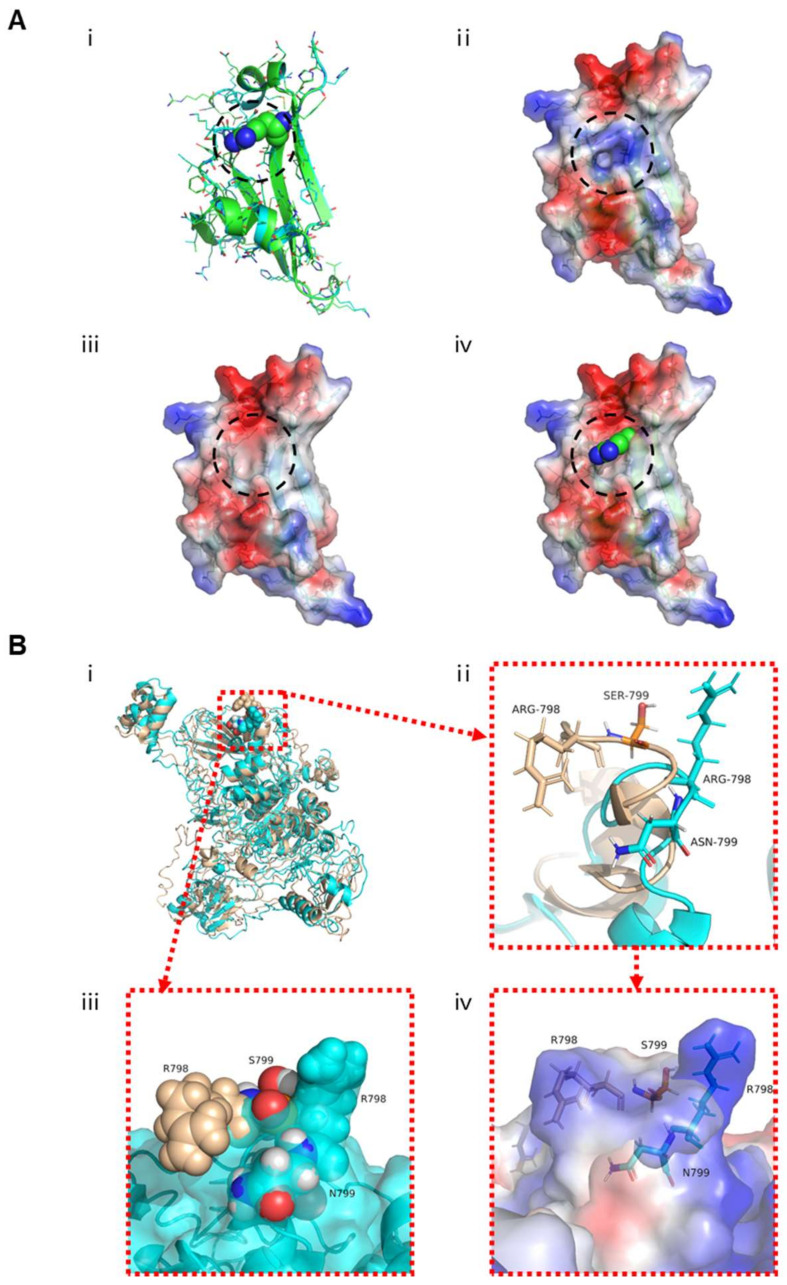
Effects of *USP26* missense mutations on the structure of the USP26 protein. (**A**) The structure comparison of wild-type USP26 and the mutant Arg825Gly. (i) Structure comparison in cartoon images (Arg825 in spheres, wild-type in cyan, and mutant in green); (ii) MEP (molecular electrostatic potential) of the wild-type USP26 structure, (iii) MEP of the mutant Arg825Gly structure; (iv) Arg825 superposition on the MEP of the mutant Arg825Gly. The mutants in the surface area are highlighted with dashed circles. (**B**) The structure comparison and close-up view of the mutant Asn799Ser. (i) Superposition and comparison of wild-type USP26’s (in cyan) whole structure and its mutant Asn799Ser (in wheat). According to the close-up view of the helix-loop-helix (HLH) structure (ii), the surface comparison (iii), and the MEP comparison (iv) of the two structures, Asn799 is located in an HLH structure motif and exposed at the surface of loop; thus, its mutant Asn799Ser tends to induce the HLH conformation change. The side chain of its neighbor residue, Arg798, has a large reorientation, which may lead to a significant surface change, as well as the electrostatic map difference.

*USP26* (NM_031907.3) is located on chromosome X, and encodes a predicted 913-amino acid protein. USP26 is specifically expressed in the testis according to the Human Protein Atlas, and is described to be associated with the deubiquitination process. Importantly, the residues in USP26 affected by these aforementioned variants are all highly conserved across species (Figure 1B). Further structural analysis by online bioinformatic tools revealed the severe effects of these amino acid-substituting mutations on the structure of USP26, including the likely changes in the specificity of the surface for binding in the USP26 mutant p.Arg825Gly, and the possible difference of the helix-loop-helix conformation or electrostatic map difference in the USP26 mutant p.Asn799Ser (Figure 2). These findings suggest that the asthenoteratozoospermia phenotypes were likely caused by the identified hemizygous *USP26* variants.

### 3.2. Hemizygous Variants in USP26 Lead to Obviously Reduced Expressions of USP26 mRNA and Proteins

**Figure 3:**
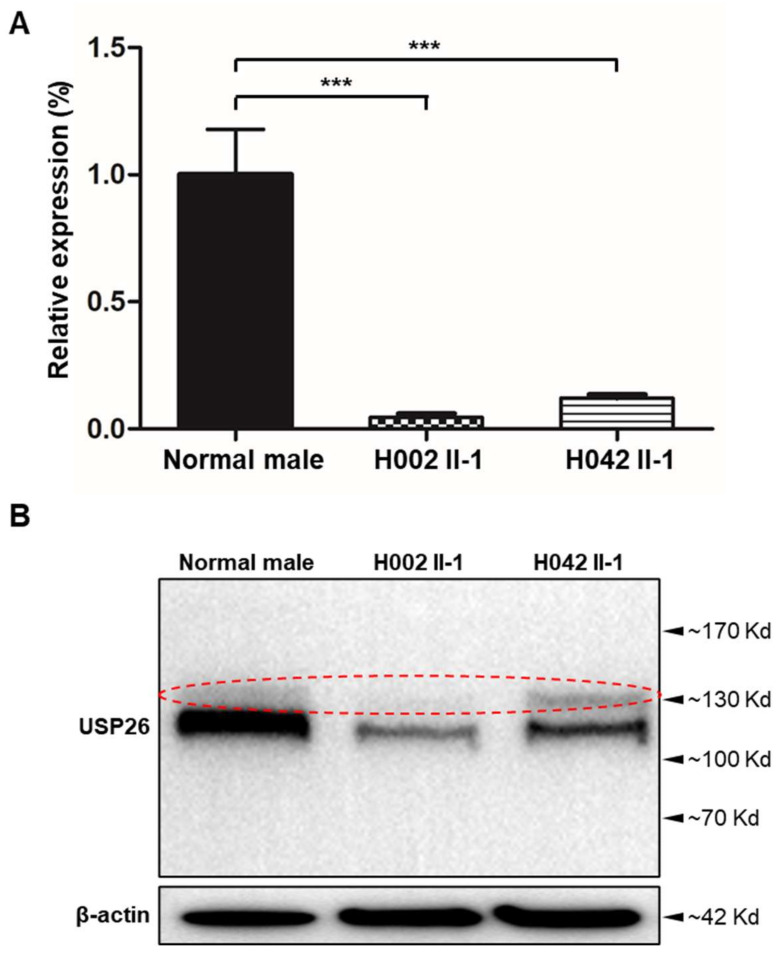
Expression analysis of *USP26* mRNA and USP26 in the spermatozoa from a normal male control and men harboring hemizygous *USP26* variants. (**A**) Real-time quantitative PCR analysis indicated that the abundance of *USP26* mRNA was significantly reduced in the sperm from men harboring hemizygous *USP26* variants, when compared to that of a normal control man. Data represent the means ± standard error of measurement of three independent experiments. Two-tailed Student’s paired or unpaired t-tests were used as appropriate (*** *p* < 0.001). (**B**) Immunoblotting assay indicated dramatically reduced expression of USP26 in the spermatozoa from men harboring hemizygous *USP26* variants. β-actin was used as a loading control. The red circle represents non-specific bands.

**Figure 4:**
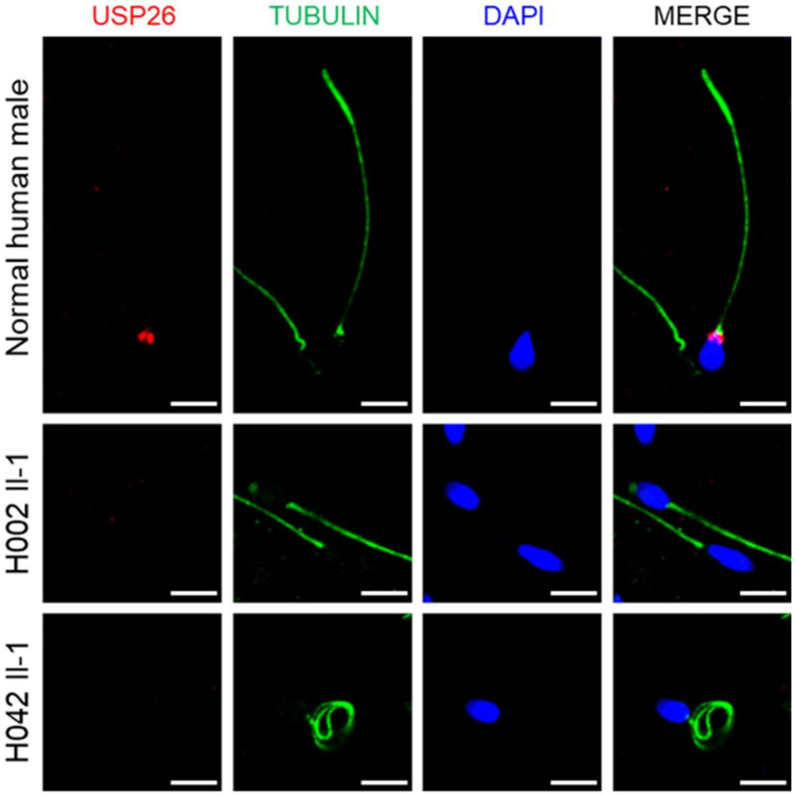
Immunofluorescence staining of USP26 in the spermatozoa from a normal male control and men harboring hemizygous *USP26* variants. The sperm cells were stained with anti-USP26 (red) and anti-a-tubulin (green) antibodies. DNA was counterstained with DAPI (4′,6-diamidino-2-phenylindole) as a marker of the cell nucleus. Scale bars: 5 mm.

To further investigate the pathogenicity of hemizygous *USP26* variants, we obtained new sperm samples from a fertile control man and from men harboring *USP26* variants. The expressions of *USP26* mRNA and protein were investigated using RT-qPCR and immunoblot assays, respectively. The abundance of *USP26* mRNA in the sperm from subjects harboring hemizygous *USP26* variants was significantly reduced when compared to the normal control (Figure 3A). As for the protein level, the expression of the USP26 protein was also obviously decreased in the sperm samples from men harboring hemizygous *USP26* variants (Figure 3B). Consistently, as shown by immunostaining assay, USP26 immunostaining was mainly localized at the base of the sperm flagella in normal spermatozoa, but was almost absent in the spermatozoa from men harboring hemizygous *USP26* variants (Figure 4). These experimental observations further indicated the important contribution of these hemizygous *USP26* variants to asthenoteratozoospermia.

### 3.3. Asthenoteratozoospermia Phenotypes in Men Harboring Hemizygous USP26 Variants

**Figure 5:**
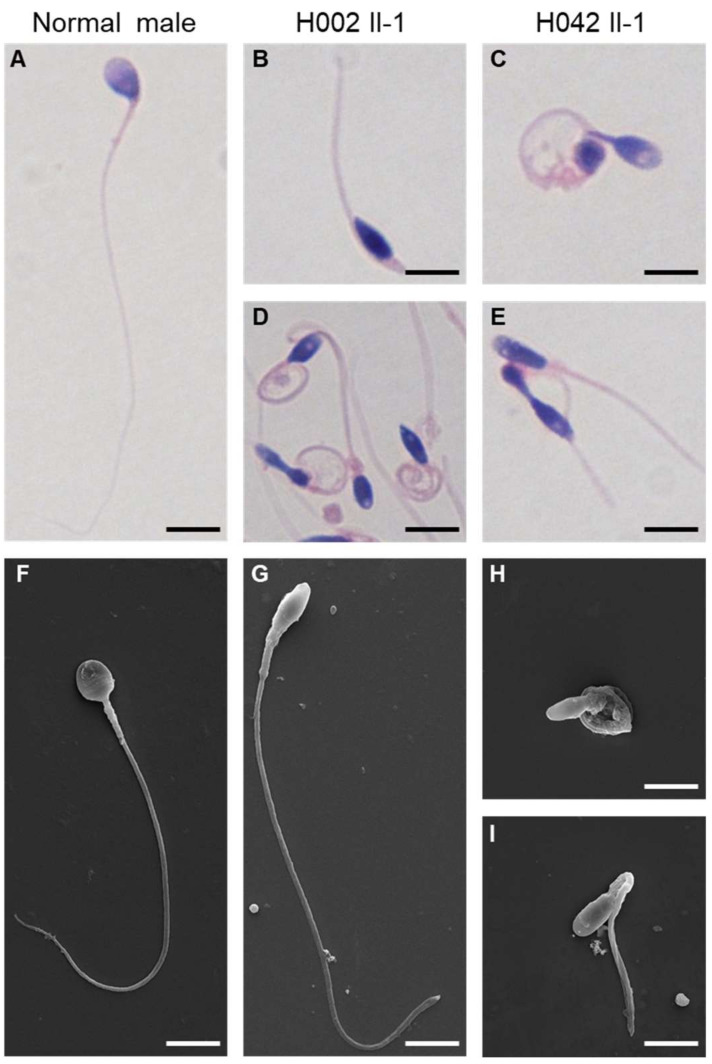
Sperm morphology analyses for men harboring hemizygous *USP26* variants. Normal morphology of the spermatozoon from a healthy control male, as revealed by light microscopy (**A**) and scanning electronic microscopy (**F**). For the spermatozoa from men harboring hemizygous *USP26* variants, multiple malformations were observed, including short (**B**,**I**) and coiled flagella (**C**,**D**,**H**), and thin heads (**D**,**E**,**G**–**I**). Scale bars: 5 mm.

**Figure 6:**
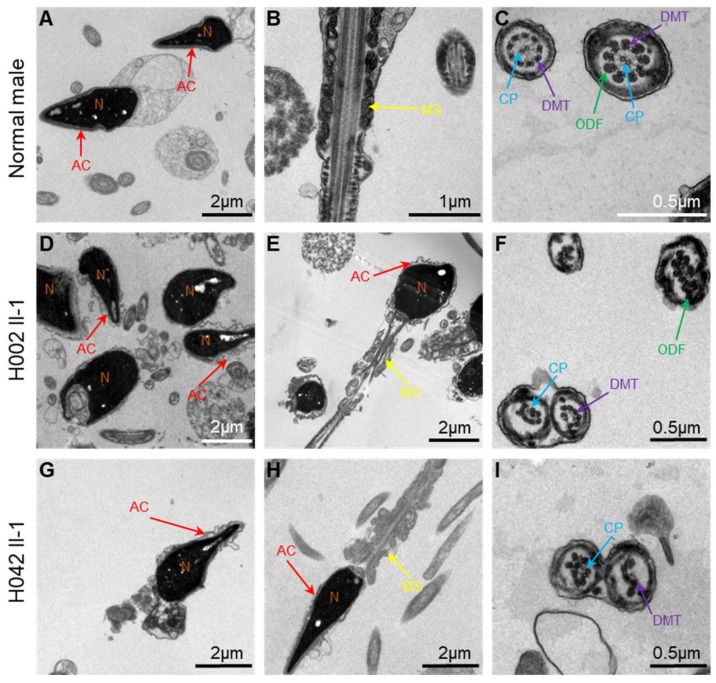
TEM analyses of sperm cells from a normal male control and men harboring hemizygous *USP26* variants. In the cross-sections of the sperm cells from a control individual, acrosomes can be seen tightly attached to the nucleus (**A**), the mitochondrial sheath is regularly distributed in the midpiece of the sperm flagella (**B**), and typical “9 + 2” microtubule structures are clearly observed (**C**). Cross-sections of the spermatozoa from men harboring hemizygous *USP26* variants revealed various abnormal ultrastructures of sperm heads and flagella, including partial defects or loss of the acrosome (**D**,**E**,**G**,**H**), disorganization of the mitochondrial sheath and peripheral microtubules (**E**,**F**,**H**,**I**), and a lack of the central pair of microtubules or other peri-axonemal structures (**F**,**I**). Abbreviations: CP, central pair of microtubules (blue arrows); DMT, peripheral microtubule doublet (purple arrows); ODF, outer dense fiber (green arrows); MS, mitochondrial sheath (yellow arrows); AC, acrosome (red arrows); N, nucleus (orange arrows).

The semen parameters of men with hemizygous *USP26* variants were investigated in the source laboratories, according to World Health Organization guidelines. Semen analysis indicated slightly reduced sperm progressive motility in both of the men harboring hemizygous *USP26* variants. A sperm morphological study using H&E staining and SEM indicated multiple malformations in the spermatozoa from men harboring hemizygous *USP26* variants, including absent, short, and coiled flagella, and thin heads (Figure 5). The rates of coiled flagella and thin heads were obviously higher in men with hemizygous *USP26* variants than in normal controls (Table 2). Furthermore, TEM was conducted to investigate the ultrastructure of spermatozoa in the subjects with *USP26* variants. As shown in Figure 6, partial defects or loss of the acrosome can be observed in the sperm heads of subjects with hemizygous *USP26* variants. As for sperm flagella, the cross-sections displayed typical ‘9 + 2’ microtubule structure (nine peripheral microtubule doublets and a central pair of microtubules) in a normal male, but presented with a higher rate of disorganization in the axonemal or other peri-axonemal structures (e.g., loss or displaced mitochondrial sheath) in the spermatozoa from men harboring hemizygous *USP26* variants (Figure 6 and Figure S1).

### 3.4. Good Prognosis of ICSI in Men Harboring Hemizygous USP26 Variants

Previous studies have suggested intracytoplasmic sperm injection treatment as an effective way to rescue asthenoteratozoospermia-associated infertility phenotypes [24]. In this study, both of the men harboring *USP26* mutations had undergone assisted reproductive therapy by ICSI treatment. As shown in Table 3, for H002 II-1, four oocytes were retrieved at metaphase II, and four were fertilized. From these, three good quality 8 cells developed, and two were used for embryo transfer. The couple achieved a single successful pregnancy and delivery. For H042 II-1, 10 oocytes were retrieved at metaphase II, and seven were fertilized. A good prognosis was also acquired after embryo transfer. Thus, ICSI can be recommended for *USP26*-associated asthenoteratozoospermia.

## 4. Discussion

Spermatogenesis is a testis-specific and multistep biological process, which is regulated by complex mechanisms. During spermatogenesis, deubiquitination enzymes play an important role in regulating diverse cellular activities and protein turnover (e.g., replacement of histones by protamine, germ cell apoptosis, mitotic proliferation, and differentiation of spermatogonial stem cells) [25,26,27,28,29]. Here, our genetic analyses using WES on a cohort of 150 cases with asthenoteratozoospermia identified two unrelated men carrying hemizygous variants in *USP26*, which is a member of the DUB family that is preferentially expressed in the testis. These *USP26* variants are either rare or absent in human populations, and were predicted to be damaging by multiple bioinformatics software programs. Further pathogenicity analyses by RT-qPCR and immunoblotting showed that the expression of USP26 was significantly reduced in the spermatozoa from men harboring hemizygous *USP26* variants. Therefore, the asthenoteratozoospermia-associated phenotypes in these cases are likely to be caused by hemizygous variants in *USP26.*

*USP26* is a single-exon gene located on the X chromosome that was first identified by Wang et al. and thought to be a retrogene that originates from autosomal Usp39/USP39 [30,31]. Previous studies have indicated that USP26 is mainly located at spermatogonia (types A and B), preleptotene and leptotene-zygotene spermatocytes, round spermatids, and the blood–testis barrier in both mouse and human testes [31,32]. Several studies have reported the association of *USP26* with male infertility. Stouffs and colleagues identified the presence of *USP26* mutations in patients with various histological patterns of spermatogenic defects (Sertoli-cell-only syndrome and maturation arrest) [16]. Genetic polymorphisms further verified the association of *USP26* with abnormal spermatogenesis, from Sertoli-cell-only syndrome to non-obstructive azoospermia or asthenoteratozoospermia [16,18,33,34,35,36,37,38]. Several spermatogenesis-associated proteins, such as the androgen receptor MDM2, SMAD7, and polycomb repressive complex 1, have been identified as substrates of USP26 [39,40,41,42,43]. In addition, *Usp26* mutation in mice also leads to defective spermatogenesis, which manifests as a decreased sperm count and a malformed sperm head morphology [44]. However, there are also studies using enzymatic assays or meta-analyses that do not support a direct association between *USP26* variants and male infertility [45,46]. These differences may be due to different ethnic origins or number of patient samples, different analytical methods, or different definitions of infertility [36]. In our study, genetic analysis by whole-exome sequencing identified two hemizygous variants in *USP26* from 2 of 150 individuals affected by asthenoteratozoospermia. The spermatozoa from men harboring *USP26* mutations also displayed multiple malformations, including higher rates of coiled flagella and thin heads. TEM analysis further revealed partial defects or loss of the acrosome, and dramatic disorganization in axonemal or other peri-axonemal structures in the spermatozoa from men harboring hemizygous *USP26* variants. Thus, our study, combined with previous findings, fully confirmed that *USP26* might be an important candidate gene for asthenoteratozoospermia.

As an assisted reproductive technology, ICSI has become an important tool for helping infertile couples achieve a successful pregnancy. Previous studies and our recent works have revealed the outcomes of ICSI for a series of asthenoteratozoospermia-related genes. For example, good clinical outcomes can be achieved by ICSI treatment using the spermatozoa from asthenoteratozoospermia-affected individuals with *DNAH1*, *DNAH8*, *TTC29*, and *CFAP47* variants [6,10,24,47]. However, only failed pregnancies were reported for *CEP135* or *DNAH17*-associated asthenoteratozoospermia, due to abnormal centriole assembly or other unknown reasons [48,49]. In our study, both of the subjects harboring *USP26* mutations underwent ICSI treatment with their own sperm, and successful clinical pregnancies were acquired, indicating that ICSI can be recommended for *USP26*-associated asthenoteratozoospermia.

## 5. Conclusions

In conclusion, our genetic and functional analyses in human subjects suggest that hemizygous variants in *USP26* are a crucial genetic cause of asthenoteratozoospermia. Our experimental observations, together with previously reported evidence in *Usp26* mutant mice, strongly support the importance of USP26 in spermatogenesis. A good pregnancy outcome can be acquired by ICSI treatment using the spermatozoa from men harboring hemizygous *USP26* variants. These findings provide new knowledge for genetic counselors and clinicians to further understand the genetic etiology of asthenoteratozoospermia and establish effective interventions.

## Figures and Tables

**Table 1:** Hemizygous deleterious *USP26* variants identified in Chinese men with asthenoteratozoospermia.

*USP26* Variant	M1	M2
cDNA alteration ^a^	c.2473C>G	c.2396A>G
Variant allele	hemizygous	hemizygous
Protein alteration	p.Arg825Gly	p.Asn799Ser
Variant type	missense	missense
Allele Frequency in Human Population		
1000 Genomes Project	0	0
East Asians in gnomAD	0.0001444	0.00007219
All individuals in gnomAD	0.00001093	0.000005463
Function Prediction		
SIFT	damaging	damaging
PolyPhen-2	damaging	damaging
M-CAP	damaging	damaging
CADD ^b^	18.76	11.84

^a^ The NCBI reference sequence number of *USP26* is NM_031907.3. ^b^ Variants with CADD values greater than 4 are considered to be deleterious.

**Table 2:** Semen characteristics and sperm morphology in men harboring hemizygous *USP26* variants.

Subject	H002 II-1	H042 II-1	Reference Limits
Semen Parameter			
Semen volume (mL)	2.4	1.6	1.5 ^a^
Sperm concentration (10^6^/mL)	31.2	31.5	15.0 ^a^
Motility (%)	31.0	51.9	40.0 ^a^
Progressive motility (%)	26.0	26.8	32.0 ^a^
Sperm Morphology			
Thin head (%)	23.0	52.8	14.0 ^b^
Absent flagella (%)	1.8	2.5	5.0 ^b^
Short flagella (%)	7.5	2.3	1.0 ^b^
Coiled flagella (%)	18.0	29.0	17.0 ^b^
Angulation (%)	1.0	2.0	13.0 ^b^
Irregular caliber (%)	1.3	1.0	2.0 ^b^

^a^ Reference limits according to the WHO standards. ^b^ Reference limits according to the distribution range of morphologically normal spermatozoa observed in 926 fertile individuals.

**Table 3:** Clinical outcomes of ICSI cycles using the spermatozoa from men harboring hemizygous *USP26* variants.

Subject	H002 II-1	H042 II-1
Male age (year)	30	34
Female age (year)	31	32
Number of ICSI cycles	1	1
Number of oocytes injected	4	10
Number (and rate) of fertilized oocytes	4 (100%)	7 (70%)
Number (and rate) of cleavage embryos	4 (100%)	7 (100%)
Number (and rate) of 8 cells	3 (75%)	3 (42.9%)
Number of transfer cycles	1	1
Number of embryos transferred per cycle	2	2
Implantation rate	100%	50%
Clinical pregnancy rate	100%	100%
Miscarriage rate	0%	0%

## Data Availability

Publicly available datasets were analyzed in this study. This data can be found here: CADD, https://cadd.gs.washington.edu/snv; 1000 Genomes Project, http://www.internationalgenome.org; gnomAD, https://gnomad.broadinstitute.org; Human Protein Atlas, https://www.proteinatlas.org; I-TASSER server, http://zhanglab.ccmb.med.umich.edu/I-TASSER;M-CAP: http://bejerano.stanford.edu/MCAP/; National Center for Biotechnology Information (NCBI), https://www.ncbi.nlm.nih.gov/; PolyPhen-2, http://genetics.bwh.harvard.edu/pph2/; SIFT, https://sift.bii.a-star.edu.sg; SWISS-MODEL, https://swissmodel.expasy.org/.

## Supplementary Materials

The following are available online at https://www.mdpi.com/article/10.3390/cells10071594/s1, Table S1: Primers used for amplification and verification of *USP26* mutations; Table S2: Primers used for the RT-qPCR assay; Figure S1: The rates of flagellar ultrastructural abnormality in the spermatozoa from a normal male control and men harboring hemizygous *USP26* variants.
