# Supplementary material for: Novel Mutations in X-Linked, USP26-Induced Asthenoteratozoospermia and Male Infertility
Source: Cells. 2021 Jun 25;10(7):1594. doi: 10.3390/cells10071594 (PMC8307012; doi:10.3390/cells10071594)
Supplement: Supplementary file 1 [file cells-10-01594-s001.zip › cells-1205964-supplementary.pdf]

Table S1. Primers Used for Amplification and Verification of *USP26* Mutations

Primer Names	Primer Sequences (5'-3')	Tm
M1-F	CTCCTGGATACCTAACACC	48°C
M1-R	CAAAGAACCTCCTAAGACC	
M2-F	GCCTCCTGGATACCTAAC	49°C
M2-R	CAAACAGTGCCCGAAA	

Table S2. Primers Used for RT-qPCR Assay

Primer Names	Primer Sequences (5'-3')	Tm
H-*USP26*-F	CAAAGAACCTCCTAAGACC	60°C
H-*USP26*-R	AGATGGCTGACAACACTAA	
H-*GAPDH*-F	GGAGCGAGATCCCTCCAAAAT	60°C
H-*GAPDH*-R	GGCTGTTGTCATACTTCTCATGG	

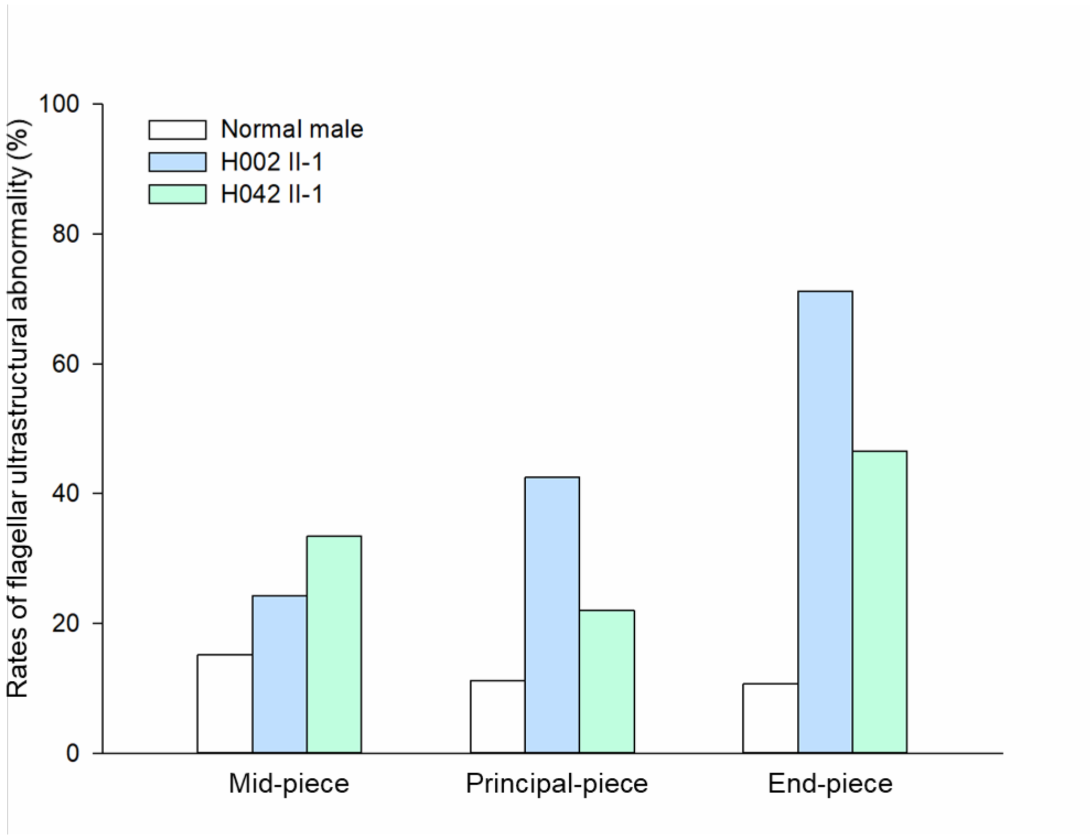

Figure S1. The rates of flagellar ultrastructural abnormality in the spermatozoa from a normal male control and men harboring hemizygous *USP26* variants. For each case, more than one hundred cross sections were counted for the analysis of abnormal ultrastructure.
